# Adipose Tissue Segmentation after Lung Slice Localization in Chest CT Images Based on ConvBiGRU and Multi-Module UNet

**DOI:** 10.3390/biomedicines12051061

**Published:** 2024-05-10

**Authors:** Pengyu Lei, Jie Li, Jizheng Yi, Wenjie Chen

**Affiliations:** 1 College of Computer and Information Engineering, Central South University of Forestry and Technology, Changsha 410004, China; 20221100369@csuft.edu.cn (P.L.); ckeiko@163.com (W.C.); 2 Yuelushan Laboratory Carbon Sinks Forests Variety Innovation Center, Changsha 410000, China

**Keywords:** deep learning (DL), localization, segmentation, subcutaneous adipose tissue (SAT), visceral adipose tissue (VAT)

## Abstract

The distribution of adipose tissue in the lungs is intricately linked to a variety of lung diseases, including asthma, chronic obstructive pulmonary disease (COPD), and lung cancer. Accurate detection and quantitative analysis of subcutaneous and visceral adipose tissue surrounding the lungs are essential for effectively diagnosing and managing these diseases. However, there remains a noticeable scarcity of studies focusing on adipose tissue within the lungs on a global scale. Thus, this paper introduces a ConvBiGRU model for localizing lung slices and a multi-module UNet-based model for segmenting subcutaneous adipose tissue (SAT) and visceral adipose tissue (VAT), contributing to the analysis of lung adipose tissue and the auxiliary diagnosis of lung diseases. In this study, we propose a bidirectional gated recurrent unit (BiGRU) structure for precise lung slice localization and a modified multi-module UNet model for accurate SAT and VAT segmentations, incorporating an additive weight penalty term for model refinement. For segmentation, we integrate attention, competition, and multi-resolution mechanisms within the UNet architecture to optimize performance and conduct a comparative analysis of its impact on SAT and VAT. The proposed model achieves satisfactory results across multiple performance metrics, including the Dice Score (92.0% for SAT and 82.7% for VAT), F1 Score (82.2% for SAT and 78.8% for VAT), Precision (96.7% for SAT and 78.9% for VAT), and Recall (75.8% for SAT and 79.1% for VAT). Overall, the proposed localization and segmentation framework exhibits high accuracy and reliability, validating its potential application in computer-aided diagnosis (CAD) for medical tasks in this domain.

## 1. Introduction

Human health has always been a widely concerned problem. Excessive body fat is a worldwide public health problem, and extreme fat content will lead to obesity, metabolic disorders, and a decrease in life quality [1]. Human metabolism speed is closely related to adipose tissue content [2,3,4]. According to [5,6,7,8,9], excessive fat may lead to fatty liver and liver inflammation and other diseases. Kwon et al. [10] showed that most cardiopulmonary risk factors are strongly and positively correlated with SAT and VAT. The works in [11,12,13] have shown that adipose tissue content affects the success rate of organ transplantation. Heart and lung transplant recipients are more likely to have a higher risk of cardiovascular disease due to high breast fat content. Anderson et al. [14] have shown that excess fat is associated with an increased risk of primary graft dysfunction (PGD) after lung transplantation. Cho et al. [15] explored the contents of muscle and SAT in lung CT images and probed the relationship between these two contents and the survival rate of patients who have received lung transplantation. The experiments in the paper have shown that excess fat also increases mortality in lung transplant patients. The studies showed that excessive fat content in the body is harsh to human health. Traditional adipose tissue estimation methods calculate the fatty tissue by the weight value or CT images and other clinical parameters such as Body Mass Index (BMI). The literature [16,17] has shown that BMI does not accurately and precisely reflect the mass of fat tissue in the body. Therefore, we need a more intelligent and accurate way to estimate human adipose tissue accurately.

Computer-aided diagnosis (CAD) has been extensively studied and applied in the medical field [18,19]. In medical image research, many researchers conducted analyses based on X-ray images. Bottigli et al. [20], when studying breast cancer, used the distributed database to analyze mammography images and classified fat and other tissues through the obtained texture analysis results. In recent years, the rapid development of artificial intelligence (AI) has also made significant progress in applying DL in the medical domain. Deep learning (DL) methods have recently drastically affected medical imaging, revolutionizing this field. Mina et al. [21] proposed a Dense Residual UNet (DRU), which made an improvement on the Residual Network (ResNet) and the Dense Network (DenseNet) to complete the medical image segmentation task. This work has superior performance in labeled datasets with few pixels and few training samples, but its utility in datasets with more examples needs to be proved. Zhang et al. [22] proposed a weak medical image segmentation supervision method. They achieved the segmentation results via the scale constraint method but compared it with the supervised model. Usman et al. [23] conducted a three-dimensional segmentation of lung CT using an adaptive region of interest (ROI) algorithm to study the exact quantification of pulmonary nodules based on the deep residual UNet structure. Still, it has yet to be reliably demonstrated for the fat segmentation task, which is more complex, irregular in shape, and more difficult to distinguish from adjacent tissues. Peng et al. [24] conducted a texture analysis on CT images. They introduced a multi-perception model to analyze further the five parameters of the lowest density value, energy, kurtosis, low-density enhancement value, and hara variance to improve the accuracy of diagnosis and reduce the misdiagnosis rate.

Adipose tissue is characterized by a complex structure, significant individual differences, and random shape [25]; image-based fat quantification, therefore, remains a considerable challenge. Cao et al. [26] proposed an unsupervised clustering segmentation method of fat based on MRI images of the human abdomen. However, due to the limitations of the unsupervised method, the distinction between SAT and VAT becomes more difficult without the guidance of labels during the training process, and there is still room for improvement in the segmentation accuracy. Hussein et al. [27] used gradient points to provide the initial boundary of SAT and VAT and then refined the edge based on appearance and geometric calculation. They finally obtained the final boundary and segmentation results through the context label fusion based on the conditional random field (CRF), which replaced the function of supervised methods with unsupervised methods. Irmak et al. [28] proposed an affinity propagation clustering method based on fuzzy connectivity (FC) image segmentation for automatic segmentation. This is considering the heterogeneity of adipose tissue but might be a bit subjective as the automated selection approach evades the need for filtering systems. Amer et al. [29] proposed a patch integration algorithm that fuses the contextual information of adjacent pixels for the irregular problem of adipose tissue and applied the UNet structure and K-Means algorithm of embedded parts to achieve the segmentation task. It has not been demonstrated whether the results obtained by this work in leg muscle and adipose tissue segmentation are equally applicable to the structurally complex task of adipose tissue segmentation in lung slices.

Estrada et al. [25] implemented image segmentation through a two-dimensional, complete convolutional network based on UNet. They competed for the output of each pooling layer and the input of its peer layer in the upsampling process after a fusion via a convolutional layer, retained the optimal one, and transmitted it to the next layer to obtain more accurate segmentation results. This network structure has yet to be demonstrated in adipose tissue segmentation in lung slices. A Squeeze-and-Excitation (SE) block is proposed for zonal prostate segmentation [30]. Koitka et al. [31] added multi-resolution blocks combined with the convolution results of adjacent layers. They obtained segmentation predictions with more comprehensive features. Langner et al. [32] compared two complete convolutional neural network structures, UNet and VNet, in a study of abdominal adipose tissue segmentation and verified the advantages of UNet. Our paper adopts the UNet structure as the baseline model and explores its performance in adipose tissue 2D segmentation. Masoudi et al. [33] used the conditional generated network (c-GAN) to generate the synthesized CT images (s-CT) of adipose tissue in the study on abdominal adipose tissue and then used the UNet structure to refine SAT and VAT s-CT. The effect of the model varies with the data, but the advantages of UNet are clear.

The fusion of spatial and learning information with contextual information can help to localize pulmonary tissues. The gated recurrent units (GRUs) [34] model works based on the recursive updating of the features in the system. Bidirectional GRU (BiGRU) is a modified GRU. It can not only preserve the learned information but also provide feedback on the future learned information and is implemented in a two-way information fusion mechanism.

In this paper, we propose a model inspired by BiGRU and a multi-module combined approach for lung slice localization and adipose tissue segmentation using the UNet structure. Compared to conventional methods in terms of accuracy and specificity, our proposed methods demonstrate superiority. The model utilizes multi-modules and attention mechanisms [35] to segment VAT and SAT in CT slices, showing a significantly better performance than feature fusion across multiple components. We explore different segmentation results of various modules (the competitive module, multi-resolution module, and attention mechanism) and the segmentation outcomes of combined modules. The results indicate more accurate segmentation of fat content values and reduced measurement errors, demonstrating a more comprehensive characterization of adipose tissue.

## 2. Materials and Methods

In this section, we introduce the overall framework and detailed design of this work, which is primarily divided into three parts: the preprocessing module, the lung slice localization model, and the adipose tissue segmentation model.

### 2.1. Overall Framework of the Proposed Methodology

**Figure 1:**
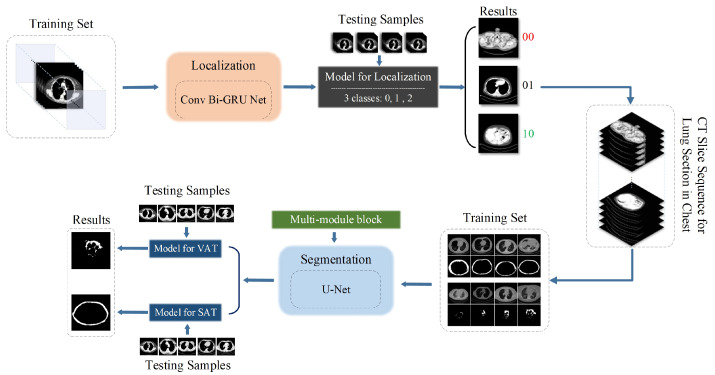
Workflow of the proposed methodology. The upper part of the diagram represents the localization model, while the lower part depicts the segmentation model. The localization model identifies the first and last slices of the lung in the CT sequence through multi-class classification, establishing the spatial location of the lung region within the CT sequence. In the results of the upper part, ‘00’, ‘01’, and ‘10’ correspond to the top slice, the bottom slice, and other slices between these boundaries, respectively. Subsequently, SAT and VAT are segmented and identified by integrating multi-modules into the layers of the UNet network. This enhancement results in improved segmentation accuracy compared to traditional UNet methods.

The overall workflow and the connection to each module are shown in Figure 1. We set the continuous lung CT slice sequences as the input in this paper.

In our approach, every five slices (this sequence size is discussed in the comparison experiment) are grouped, taking into account their positional relevance and order in the body. For each sample, the continuous CT image sequences’ information is fused to automatically position the lung in CT images based on BiGRU. The localization task essentially involves a triple-classification process. The resulting three categories (the first slice of the lung, the last portion of the lung, and other pieces, i.e., FL, LL, and OS) are used to locate the entire lung, thereby determining the spatial location of the CT sequence of the lung. Lung slice localization serves as a selection step to exclude unnecessary slices in the whole chest CT for the subsequent segmentation process.

The input of the segmentation model is chosen from FL to LL (including the pieces in between) and ultimately detects the SAT and VAT areas using the multi-module UNet model. Additionally, in this paper, we test the combination of multi-module mechanisms (i.e., multi-resolution, the competitive module, and the attention module) used to modify the original UNet, which leads to improvements in the segmentation accuracy in the localized lung CT slices.

### 2.2. Lung Slices Localization Model in Chest CT Imaging

**Figure 2:**
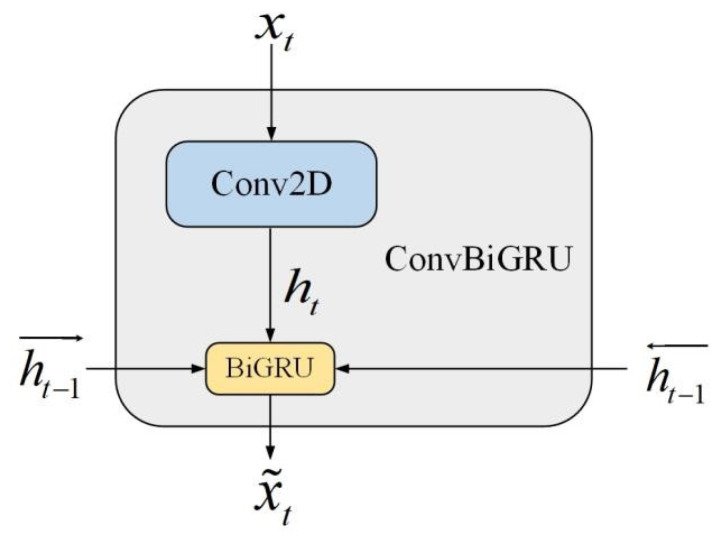
ConvBiGRU structure. The input is the CT slice sequence with five slices in each group, and the output is the specific class of the slices. There are overall three class results of the output in localization. The $\vec{h_{t-1}}$ and $\overset{\leftarrow }{h_{t-1}}$ represent the forward and backward information, respectively, and ${\tilde{x}}_{t}$ is the obtained results at time *t*.

Observing the CT slice sequence, the lung is consistently surrounded by intricate tissues, with variations observed from case to case. Distinguishing the targeted slice from its neighboring pieces presents a challenge, even for seasoned experts, due to the overall variability among organs and imaging conditions. This challenge is further compounded in cases where differentiating nodules from tumors is necessary. This study underscores the enhancement of pulmonary localization results through the amalgamation of structure and texture information from lung CT slices. The localization task is tackled through the fusion of contextual information within the CT slice sequence. To accomplish this, the ConvBiGRU model, depicted in Figure 2, performs the fusion of information from multiple slices along with their spatial correlation.

In this model, the original linear layers are replaced by convolution layers to extract the spatial image information. The input of ConvBiGRU requires over three CT slices due to the space contextual information need, which will be discussed in the comparison experiment part. The model extracts characteristic information of these slices through forwarding and backpropagation. The presented model is inspired by the ConvLSTM network [36], combining forward and backward spatial information with temporal information. The convolution part reduces the spatial redundancy while the BiGRU function extracts more comprehensive information than LSTM and finally precisely identifies the slices with the lung. The update gate, $z_{t}$, at the time *t* in a GRU block is as follows:

(1) $$z_{t}\;=\;\sigma \left(W_{z}\cdot \left[h_{t-1},x_{t}\right]\right)$$

where $h_{t-1}$ and σ are the information at t−1 and activation functions, respectively. The update gate determines the amount of information retained from t−1. The reset gates at time *t* in the model, $r_{t}$, are as follows:

(2) $$r_{t}\;=\;\sigma \left(W_{r}\cdot \left[h_{t-1},x_{t}\right]\right)$$

The reset gate is considered to be a linear transform of the combined information at the time t−1 for the new input with a sigmoid activation function.

(3) $$\tilde{h_{t}}\;=\;\tanh\;\sigma \;\left(W\cdot \left[r_{t}\cdot h_{t-1},x_{t}\right]\right)$$

Let ${\tilde{h}}_{t}$ be the updating value of the gate, where $\;{\tilde{h}}_{t}$ represents the newly generated information from the reset gate, $r_{t}$, while activated by a hyperbolic tangent function. Adding the results from two entrances, the output of the model obtained by the following recursive function at time *t* is as follows:

(4) $$h_{t}\;=\;\left(1-z_{t}\right)\cdot h_{t-1}+z_{t}\cdot \tilde{h_{t}}$$

**Figure 3:**
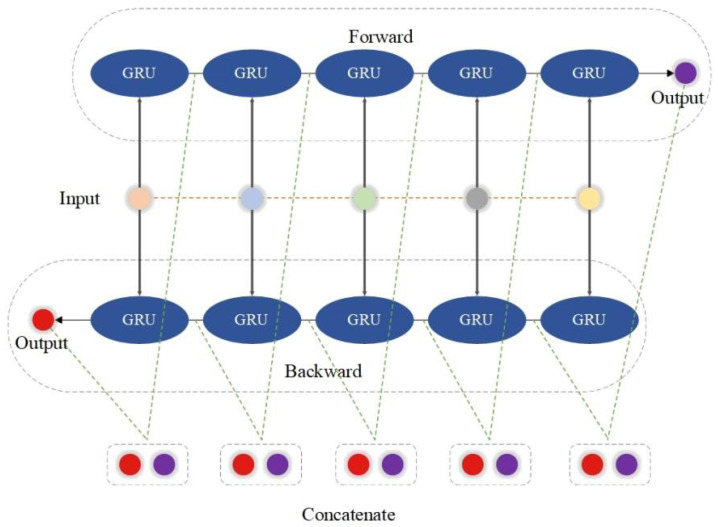
Schematic diagram of BiGRU. It mainly contains two different directions of GRU with the same input sequence and a concatenation of the two outputs in the end. The input circles with different colors in the middle belong to one group of the input sequence.

Like the GRU model, the BiGRU operates using the same logic but multiple times in two different directions, as presented in Figure 3.

The model fuses forward information and receives information from the back. To perform a lung localization, we measure the interval distance between the classification predictions and the ground truth, as well as the evaluation index of the model. As the model takes information fusion from multiple CT slices, the feature learning in the model can be affected by the length of the input sequences. To alleviate that, we performed a cross-comparative analysis of the model with different sizes of input sequences and other normalization methods. Different lengths of lines (three slices, five slices, and seven slices) were tested to compare the results to determine the optimal one.

At the same time, to minimize the loss and increase the learning effect of the model through feature intensity, we performed two different normalizations. For the first normalization, called *MaxIntensity*, we rescale the image’s pixels by the maximum power of the image, $f_{n}\left(x,y\right)=f_{n}\left(x,y\right)/4095$, where $f_{n}\left(x,y\right)$ means the pixel intensity of the image at (x,y) coordinate and 4095 represents the maximum intensity of the pixels in the image. For the second normalization way, called *MaxMinNorm*, we rescale the pixels’ intensity in the range of [0,1], as shown below:

(5) $$f_{n}\left(X,Y\right)=\frac{f\left(X,Y\right)-\min\left(f\left(X,Y\right)\right)}{\max\left(f\left(X,Y\right)\right)-\min\left(f\left(X,Y\right)\right)}$$

where min(·) and max(·) denote the same slice’s minimum and maximum pixel intensity, respectively.

Because the original dataset has massive medical images of coarse types of the lesion including bone, abdomen, lung, and five more classes, the localization part of this paper was trained on 1483 images containing the target lung region selected from 2184 CT images of 100 patients, and 252 images were randomly chosen as the test set. The validation set was separated individually from the training set with a ratio of 2:8 in the localization process to improve the model.

### 2.3. Adipose Tissue Segmentation Using Multi-Module UNet

**Figure 4:**
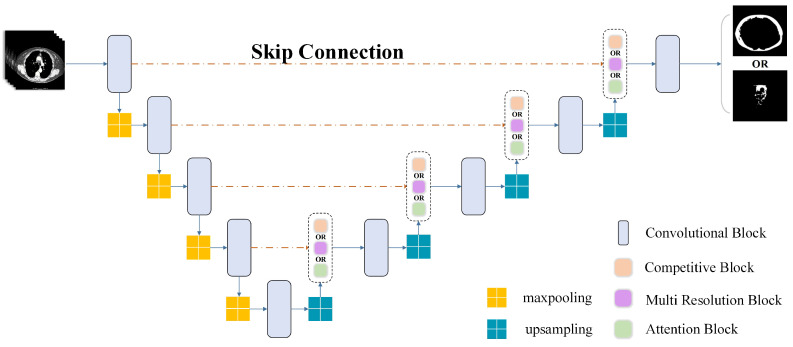
Segmentation model structure. Adding the blocks and converting the original model to the multi-module structure through the cross-test. It should be noted that attention modules only set the upsampling at the peer layer as the input.

A UNet-based multi-module model is used to segment SAT and VAT. We conduct the cross-comparison among the attention, multi-module, and multi-resolution blocks. The best combination improves the accuracy and robustness of the model to perform segmentation. The segmentation structure is shown in Figure 4.

**Figure 5:**
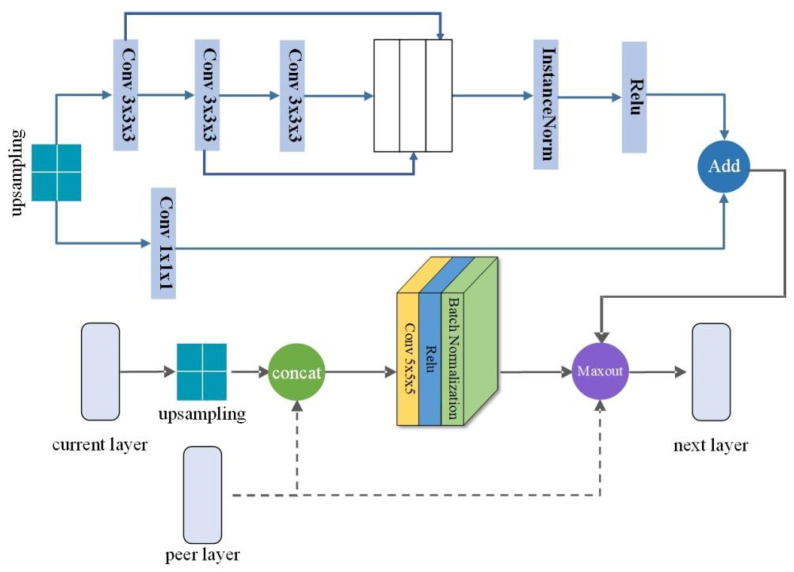
Combination structure of competitive mechanism block and multi-resolution block. The steps before the ‘Add’ operation represent the multi-resolution block, and the operations below are the competitive mechanism part. All different modules will eventually go through Maxout, using its strong fitting ability to obtain more accurate results.

Three modules, including a multi-resolution module, competitive module, and attention mechanism module, are added to the original UNet structure and compared to their performance. The output of the multi-resolution module is combined with the production of different convolutional layers and then transferred to the next layer as the input. The competitive module maxes out the result by comparing the outputs of the multi-resolution module and the peer layer. The attention mechanism reduces the influence of noise and low-intensity contrast between VAT and SAT. Through the different combinations of these modules, we conduct a comparative analysis of the segmentation. Figure 5 presents the variety of the multi-resolution module and the competitive module.

In addition to obtaining the optimal outcome between each upsampling layer and their corresponding pooling layer, the multi-resolution results are also added to the competition to construct a two-step selection mechanism. Applying the sigmoid function in the model, we achieved a binary classification for predictions, which maps the data into (0,1). The sigmoid function is defined as follows:

(6) $$S\left(p\left(x,y\right)\right)=1/\left(\exp\left(-p\left(x,y\right)\right)\right)$$

where $p\left(x,y\right)$ represents the probability value of the corresponding (x,y) coordinates. The optimizer is Stochastic gradient descent (SGD) used in the model. This algorithm makes a more effective outcome while having some level of redundancy. To maintain the training speed while the number of samples increases, each weight update in SGD updates once during its iteration. The updating process is shown as follows:

(7) $$\theta_{j}=\theta_{j}+\left(y^{i}-h_{\theta }\left(x^{i}\right)\right)x_{j}^{i}$$

where $h_{\theta }$ is the prediction to $x^{i}$, $y^{i}$ as input samples and the output of the layer, respectively, and $x_{j}^{i}$ is the *j*th variable in the *i*th sample. To accelerate SGD in the relevant direction, we use the momentum parameter beta β set to 0.9 to calculate the exponentially weighted averages as follows:

(8) $$v_{dW}=\beta v_{dW}+\left(1-\beta \right)d_{W}$$

(9) $$v_{db}=\beta v_{db}+\left(1-\beta \right)d_{b}$$

where $d_{W}$ and $d_{b}$ is the weight and bias, respectively. Having noise in the results is seemingly inevitable due to the local randomness during the descent. The loss is measured by the Dice coefficient distance/loss (DCL) and measured as follows:

(10) $$DC\left(y_{ture},y_{pred}\right)=\frac{\kappa +2\sum y_{ture}\cdot y_{pred}}{\kappa +\sum y_{ture}\cdot y_{true}+\sum y_{pred}\cdot y_{pred}}$$

(11) $$DCL\left(y_{ture},y_{pred}\right)=1-DC\left(y_{ture},y_{pred}\right)$$

where the coefficient of κ is set as 1 in the model. $y_{true}$ and $y_{pred}$ represent the ground truth and the predicted value of a CT image, respectively.

We use the weight penalty in our analysis to tackle the imbalance input sample problem for the localization model, which improves the learning convergence speed. In other words, we calculated the ratio of each sample through a logarithmic function to proportionally and effectively control the growth rate. The formula to calculate the sample weight is as follows:

(12) $$weight=weight_{i}/\sum_{i=1}^{i=n}weight_{i}$$

(13) $$W=1/\log\left(\alpha +weight\right)$$

where $weight_{i}$ is the times of occurrences of each category and *n* and α are the number of sample categories and the factor controlling the sample proportion, respectively. The constant α is set to 1.1 based on experience in the experiment to avoid negative numbers and zeros. Thus, $W\in (\frac{1}{\log2.1},\frac{1}{\log1.1})$.

Finally, the sample weight is added to the loss as follows:

(14) $$DCL_{W}\left(y_{true},y_{pred}\right)=DCL\left(y_{true},y_{pred}\right)+W$$

The segmentation part of this paper was trained on 2351 CT images, and 975 images were selected as the verification set each time, which was verified by the four-fold cross-verification method. Besides the images from the localization, we added the remaining images to the dataset to ensure sufficient training. Then, 572 and 573 images were selected as the test set for SAT and VAT segmentation, respectively. The validation set was separated individually from the training set with a ratio of 2:8 in the segmentation process to improve the model.

## 3. Results

Here we describe the experiment in detail and analyze the experimental results. This study selected 100 patients with lung CT images from the DeepLesion dataset by the National Institutes of Health Clinical Center (NIH CC) [37]. The CT images from the original dataset were all 16-bit unenhanced grayscale images (512×512×1) in png format. Slice spacing among these input CT images ranged from 0.25 mm, 0.4 mm, 0.6 mm, 1 mm, 1.25 mm, 2 mm, 2.5 mm, and 3 mm to 5 mm.

### 3.1. Data Preprocessing

For our study, we meticulously selected lung slice images from the DeepLesion dataset based on specific criteria. We focused on CT scan sequences that comprehensively captured the entire range of lung anatomy from initial appearance to disappearance. Within these sequences, we categorized the slices into distinct lung regions: apex, midsection, and base. From the original DeepLesion dataset, which encompasses diverse medical images covering various lesion types, we specifically extracted 1483 CT images containing lung regions out of a total of 2148 images from 100 patients. Our selection process aimed to ensure the representation of typical lung anatomy and pathology encountered in clinical practice. The label annotation tool [38] was used to manually label the images. Our group annotated the labels with the guidance of an expert and verified them by a professional experienced radiologist. Labels are stored and read following the serial number of the cases. Segmentation labels are recorded in JSON format, and images and their labels are saved accordingly in eight-bit *png* format.

After reading the images and their labels, we translated them into array format and split them into training and testing sets. The images and labels were augmented in 16-bit format to improve the contrast between different tissues, organs, and other components. The augmentation solely enhances contrast without cropping or rotating the images. This facilitates the manual labeling of the targeted slice and segmentation of their labels. Converting the images to eight-bit format resulted in missing information, leading to greater disparity in prediction outcomes. Therefore, we only convert them for annotation work, not for the actual training input.

**Figure 6:**
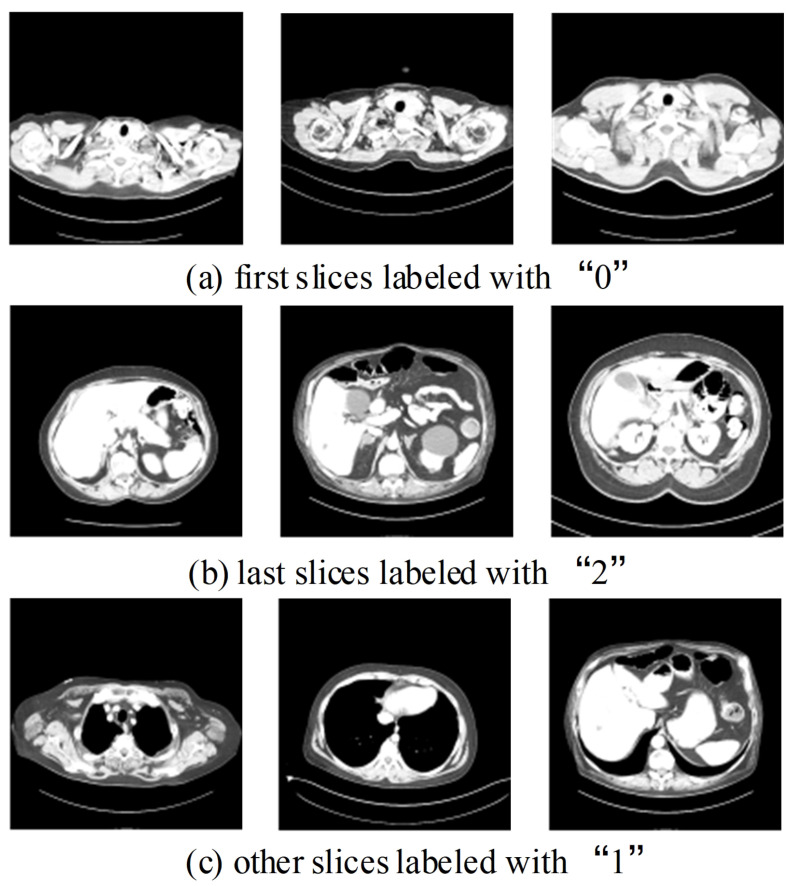
Examples of three classes of localization labels. There are large differences in the size of body tissues between different cases, which brings difficulties to the training and testing effects of the model.

We conducted a multi-class classification to localize the lung slices and labeled them based on their position in the CT image as ‘0’, ‘1’, or ‘2’ (Figure 6). ‘0’ signifies the first slice of the lung, ‘2’ denotes the last slice of the lung, and any other slices outside of these boundaries are labeled as ‘1’ (as depicted in Figure 6).

With these three categories, we could distinguish the different locations of the lung in slices. The label is processed by one-hot encoding into binary format and then input into the network.

All experiments in this study were conducted in a Python 3.6 environment, running on a computer equipped with an Intel Core™ i7-9750H CPU, 8GB RAM, and a GTX 1660TI GPU. Additionally, the computational tasks were also performed on four high-performance servers in our laboratory. These servers are equipped with NVIDIA Titan Xp series GPUs and Intel i9 processors, providing robust support for our experiments.

### 3.2. Results of Lung Slice Localization

When using the entire dataset for training and testing subsets, sample imbalance problems in localization would affect the results, i.e., much more irrelevant slices than lung slices (the first and the last slices). This will result in an imbalanced classification of the model as the features learned by the model may be more biased toward negative samples, which weakens the correct model prediction. To solve this problem, we divided the training set into three subsets with 700 slices in each subset. The total number of annotated lung slices in each subgroup was kept at 320(±10) to balance the proportion of positive and negative samples. Moreover, we add a penalty during the backward propagation according to different types of incorrect outputs in the classification model. Every error will add a weight of −1 to the misclassification in the results. We obtained more balanced results in the localization model based on this mechanism. We also conducted comparative analyses by the different number of input sequence slices (three, five, or seven slices) and normalization methods (*MaxIntensity* or *MaxMinNorm*). These experiments were conducted based on three subdatasets from the segmentation datasets on 2315 CT slices and tested on 975 CT slices. The results are presented in Table 1.

**Figure 7:**
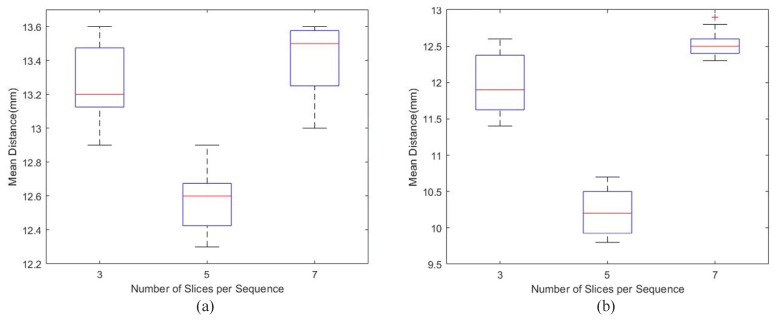
The localization model visualization results of two scheme combinations. (**a**) is the boxplot of scheme 1; (**b**) is the boxplot of scheme 2. The horizontal lines in each box in the chart represent maximum, Upper Quartile (Q3), median, Lower Quartile (Q1), and minimum, respectively, which indicate the stability and distribution of data. The statistical data in the figure come from the test results of a set of 200 slice images of the test set randomly selected, which do not completely overlap with the data selected in Table 1, which further supports the stability of the results.

As shown in Table 1, the results of *MaxMinNorm* are better than those of the *MaxIntensity* normalization method. Also, the pulmonary texture information obtained by *MaxMinNorm* is preserved better than other normalization techniques. The results of the different number of slices indicated that five slices of input yielded the best localization outcome. At the same time, three slices of input reduced the proportionality of positive and negative samples, negatively affecting localization prediction. Also, the number of negative examples in the slice sequence increased for the seven-slice input while the proportion of positive models decreased. Boxplots show the difference between the two normalization methods and input slices in Figure 7.

In Figure 7, we plotted the boxplots with the different numbers of slices per sequence under the two normalization techniques. It shows that the slice spacing obtained in *MaxMinNorm* is generally lower, and the variation range is more extensive than the *MaxIntensity* normalization technique. However, the median value of *MaxMinNorm* is more stable, and its distribution of Q1 and Q3 is more uniform. To calculate the spacing distance for the localization analysis, we used the following equation:

(15) $$d_{t}=\left|y_{pred}-y_{true}\right|\cdot sp_{t}$$

where $sp_{t}$ represents the distance between adjacent slices in this case, and $d_{t}$ represents the slice distance between the predicted result and its targeted position in the ground truth (in millimeters—mm). In the localization experiment, the average distance between the ground truth and the predicted target is shown by the Mean Distance (MD) and is calculated as follows:

(16) $$MD=\sum_{i=1}^{N}\left(\left|No_{pred}-No_{true}\right|\cdot sp_{t}\right)/N$$

where $No_{pred}$ is the number of the predicted slice with the highest score in the tested volume. $No_{true}$ is the number of the first or last slices (localizing the lung area). *N* is the total number of testing samples, and $sp_{t}$ is as mentioned before.

### 3.3. Results of Adipose Tissue Segmentation

Several comparative experiments were carried out to evaluate the performance of the segmentation model. The segmentation model can consider adipose tissues, VAT, and SAT as binary classification problems for each task. We conducted four experiments to segment both types of adipose tissue automatically. The experimental results of UNet and different modules based on UNet are shown in Table 2.

The results indicated that the overall segmentation of the SAT tissue has higher accuracy and better performance than VAT tissues using the proposed models. The model with the multi-resolution (MR) module has the best performance, while it is close to the results of the model with the attention module (ATTN). Both these two results exhibited better performance than the competitive module (CP), while the overall difference is negligible and all better than the baseline UNet model. For the segmentation of VAT, the segmentation model with MR showed the best performance, followed by the ATTN and CP models with a small gap.

We conducted a deep analysis on the comparative experiments of each module with baseline UNet. We also aggregated every two-module group to compare with the baseline UNet. Table 3 shows the experimental results of such a segmentation SAT and VAT combination.

The results indicate that the combination of the modified UNet model with MR and ATTN has the best performance in this segmentation task. Then, the modified UNet with ATTN and CP showed better accuracy than other additive modules of MR and CP. They are adding an ATTN block for the segmentation of VAT-inhibited redundancy, which brings the information of other larger thoracic tissues.

The lung CT slices showed that the adipose tissue had non-uniformity and irregularity, i.e., heterogeneity, while the adipose tissue has a similar morphological profile to fluid in the body. Comparing the prediction and ground truth visually in segmentation demonstrates the significant performance while missing some tiny fat tissues at the edge of the predicted results. The misclassification in the mentioned regions might be because of the blurred border between the pixel intensity of the adjacent area. During the experiment, we adjusted the label of the training set to clean the misclassified parts and screened the CT slices with big noises. This is aimed to ensure the relative purity of the training dataset and exclude the negative influence during the learning process that is not supposed to be there.

**Figure 8:**
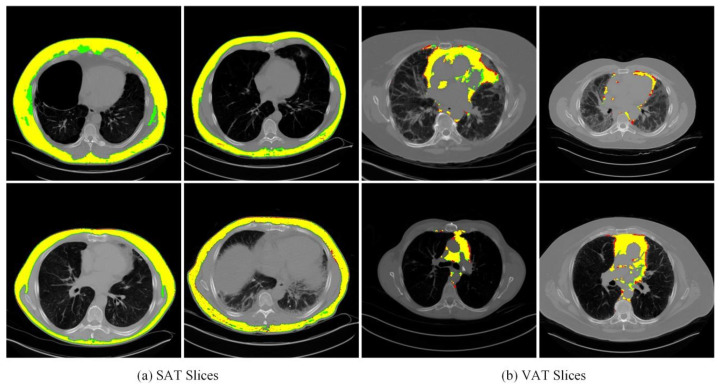
Visualizations of segmentation results. Both the segmented parts and ground truth for SAT and VAT are visualized. Green, yellow, and red regions represent the ground truth, true predictions, and false predictions, respectively.

The model applied a four-fold cross-validation to segment SAT and VAT to ensure the results’ reliability and robustness. For that, the averaged overall Precision and Recall rates were computed along with the overall averaged F1 Score and averaged Dice Score for the segmentation accuracy. Some examples of segmented parts and their corresponding ground truth for SAT and VAT are visualized in Figure 8.

The proposed segmentation model showed better accuracy in segmenting lung adipose tissue than other models [21,39,40,41,42]. The proposed model outperformed other approaches in SAT or VAT segmentation tasks (see Table 4). From Table 4, it can be observed that there are significant differences in the performance of the Dice Score, F1 Score, and Precision, while the performance of Recall shows little variation. Recall represents the proportion of samples that are truly positive and are correctly predicted as positive by the model, calculated as Recall = TP/(TP + FN). The similar Recall values across all methods in Table 4 are due to the balanced nature of our dataset and the design of our experiments. We carefully balanced the dataset during preprocessing to ensure an equal representation of positive and negative samples. Additionally, our experiments were conducted under similar conditions, including the same evaluation metrics and testing procedures. As a result, the Recall values reflect the ability of each method to correctly identify positive instances relative to the total number of positive instances in the dataset, leading to a comparable performance across methods.

## 4. Discussion

This study proposes an automated model for lung localization and adipose tissue segmentation in CT images using deep learning. The ConvBiGRU structure, incorporating contextual spatial features, is utilized for lung tissue localization. Subsequently, a UNet structure modified by a multi-resolution module and attention mechanism is employed for segmenting subcutaneous adipose tissue (SAT) and visceral adipose tissue (VAT). Contextual information, such as forward and backward spatial information, is utilized for lung slice localization. In this study, the dataset is divided into multiple subsets to address sample imbalance issues. Furthermore, comparative analyses are conducted on different image normalization methods, input numbers, and multi-module combinations. According to Table 4, it can be observed that different network methods exhibit certain differences in the segmentation performance of SAT and VAT. For example, DRUNet shows a slightly better performance than other methods in SAT’s Dice Score, F1 Score, and Precision, while lagging slightly behind in VAT’s Recall. In contrast, FedDG performs the best in SAT’s Recall but slightly lower in other metrics compared to other methods. Our method overall outperforms other methods, not only showing the best performance in SAT and VAT’s Dice Score and F1 Score but also maintaining high levels of Precision and Recall, indicating the comprehensive advantage of our method in localization and segmentation accuracy. We employ a ConvBiGRU structure for lung slice localization, combined with a multi-module UNet structure for SAT and VAT segmentation. This combination not only helps in accurately localizing lung slices but also accurately identifying SAT and VAT in segmentation tasks, reflecting the advancedness of our method in model design and structure optimization. Our method exhibits good stability in performance metrics with small deviation ranges, indicating its high robustness.

However, we also acknowledge some limitations in this study. Firstly, positive and negative classifications exist within the boundaries of adipose tissues due to the fuzzy areas surrounding other tissues. Additionally, although our model achieves satisfactory results on the current dataset, we must explicitly state that these results cannot be directly generalized to other datasets. Because different datasets may have different features and distributions, our model needs to be validated and adjusted on different datasets to ensure its generalization ability and reliability. Most importantly, our research results do not imply that our model can replace the diagnosis and judgment of professional radiologists. Although our model performs well in lung localization and adipose tissue segmentation, the expertise and experience of doctors are still indispensable in clinical applications. Our research aims to provide auxiliary diagnostic tools for doctors rather than replace their decisions.

## 5. Conclusions

To address the significant challenges associated with adipose tissue segmentation in lung CT imaging, this paper introduces an automated lung localization and adipose tissue segmentation model employing deep learning techniques. The proposed framework consists of a bidirectional gated recurrent unit (BiGRU) structure for lung slice localization and a multi-module UNet model for segmenting subcutaneous adipose tissue (SAT) and visceral adipose tissue (VAT). Furthermore, the BiGRU Net model is enhanced through the incorporation of an additive weight penalty term. In the segmentation phase, we investigate three combined mechanisms—attention, competition, and multi-resolution—within the UNet structure to assess their impact on SAT and VAT segmentation, facilitating a comparative evaluation. Finally, extensive experiments conducted on a publicly available dataset demonstrate the efficiency and effectiveness of the proposed framework.

In our future research, firstly, we plan to further optimize the model performance, especially in positive and negative classification. Although our model performs well in localization and segmentation tasks, there still exists a certain proportion of positive and negative classifications. Future work will focus on how to improve the model to reduce the occurrence of this situation, with possible methods including introducing more contextual information or exploring more complex post-processing techniques. Secondly, we will attempt to apply the model to more diverse datasets. While we conducted experiments on one publicly available dataset, we are aware that different datasets may have differences. Therefore, we plan to apply the model to more datasets from different sources and with different characteristics, further validating its robustness and universality. Lastly, we will focus on the application of the model in clinical practice. We will collaborate with radiologists to compare our model with the results manually segmented by them, further validating its feasibility and effectiveness in actual clinical settings. Through the aforementioned improvements and explorations, we believe that our research will provide a deeper understanding of adipose tissue segmentation in lung CT images and offer valuable references and insights for the development of future related studies.

## Figures and Tables

**Table 1:** Results of the localization model for lung slices.

Number of Slices in a Sequence	Mean Distance (mm)	
	***MaxIntensity***	***MaxMinNorm***
Three	13.2 ± 6.3	12.7 ± 5.7
Five	12.5 ± 4.6	10.2 ± 6.3
Seven	13.4 ± 4.4	12.5 ± 8.6

**Table 2:** Comparative results of segmentation using single-module modified UNet structures.

Model	Adipose Type	Dice Score	F1 Score	Precision	Recall
UNet	SAT	90.3 ± 0.8	81.2 ± 0.7	95.9 ± 0.9	74.6 ± 0.9
	VAT	79.8 ± 1.6	74.1 ± 1.5	77.3 ± 1.4	78.6 ± 1.9
**UNet + MR**	**SAT**	**90.7 ± 0.8**	**81.6 ± 0.8**	**96.4 ± 0.4**	**75.0 ± 0.8**
	**VAT**	**81.4 ± 1.6**	**77.1 ± 1.8**	**78.2 ± 1.8**	**77.1 ± 2.1**
UNet + ATTN	SAT	90.5 ± 1.2	81.7 ± 0.9	95.6 ± 0.4	74.3 ± 0.8
	VAT	80.8 ± 2.1	76.0 ± 2.0	76.6 ± 1.4	75.2 ± 2.4
UNet + CP	SAT	90.3 ± 0.9	81.3 ± 0.8	96.0 ± 0.6	74.5 ± 1.2
	VAT	80.5 ± 1.7	75.6 ± 1.7	76.6 ± 1.4	74.8 ± 1.8

**Table 3:** Comparative results of segmentation using multi-module modified UNet structure.

Model	Adipose Type	Dice Score	F1 Score	Precision	Recall
**UNet + MR + ATTN**	**SAT**	**92.0 ± 1.7**	**83.5 ± 1.6**	**96.4 ± 2.5**	**77.5 ± 1.2**
	**VAT**	**82.6 ± 1.4**	**78.6 ± 2.4**	**78.9 ± 2.9**	**79.3 ± 3.1**
UNet + MR + CP	SAT	91.7 ± 1.1	83.9 ± 1.3	95.9 ± 2.3	75.2 ± 2.4
	VAT	81.1 ± 1.6	76.4 ± 2.2	77.4 ± 2.3	77.3 ± 2.3
UNet + ATTN + CP	SAT	91.9 ± 0.7	83.5 ± 2.6	95.7 ± 1.7	74.8 ± 2.3
	VAT	80.6 ± 1.2	76.0 ± 3.0	76.0 ± 2.6	76.9 ± 3.1

MR: multi-resolution module; ATTN: attention module; CP: competitive module.

**Table 4:** Comparison results of segmentation with other methods on the same dataset.

Model	Adipose Type	Dice Score	F1 Score	Precision	Recall
DRUNet [21]	SAT	91.1 ± 1.1	82.0 ± 2.5	95.9 ± 1.7	72.5 ± 4.5
	VAT	80.1 ± 2.5	78.0 ± 2.7	77.3 ± 2.7	75.4 ± 3.7
UNet [39]	SAT	87.8 ± 2.7	81.5 ± 2.5	91.7 ± 0.9	74.6 ± 1.5
	VAT	81.2 ± 1.3	77.3 ± 3.4	76.1 ± 2.2	78.6 ± 2.1
UNetTransformer [40]	SAT	88.6 ± 1.5	80.9 ± 1.8	91.5 ± 1.9	76.9 ± 5.5
	VAT	81.4 ± 2.0	78.3 ± 4.9	75.7 ± 3.6	78.1 ± 4.2
FedDG [41]	SAT	90.7 ± 2.1	83.3 ± 3.5	92.2 ± 2.4	75.0 ± 1.8
	VAT	80.1 ± 2.5	73.4 ± 3.3	75.3 ± 6.4	77.1 ± 2.1
Transfuse [42]	SAT	91.3 ± 1.6	82.1 ± 2.0	95.2 ± 4.6	76.7 ± 2.7
	VAT	80.6 ± 2.9	77.4 ± 4.7	76.4 ± 2.4	77.6 ± 3.3
**Ours**	**SAT**	**92.0 ± 1.7**	**83.5 ± 1.6**	**96.4 ± 2.5**	**77.5 ± 1.2**
	**VAT**	**82.6 ± 1.4**	**78.6 ± 2.4**	**78.9 ± 2.9**	**79.3 ± 3.1**

## Data Availability

The DeepLesion datasets, which were used and analyzed during the current study, are accessible at the following URL: https://nihcc.app.box.com/v/DeepLesion, accessed on 4 April 2023.
